# Effect of Maternal Methionine Supplementation on the Transcriptome of Bovine Preimplantation Embryos

**DOI:** 10.1371/journal.pone.0072302

**Published:** 2013-08-21

**Authors:** Francisco Peñagaricano, Alex H. Souza, Paulo D. Carvalho, Ashley M. Driver, Rocio Gambra, Jenna Kropp, Katherine S. Hackbart, Daniel Luchini, Randy D. Shaver, Milo C. Wiltbank, Hasan Khatib

**Affiliations:** 1 Department of Animal Sciences, University of Wisconsin, Madison, Wisconsin, United States of America; 2 Department of Dairy Science, University of Wisconsin, Madison, Wisconsin, United States of America; 3 Adisseo USA Inc., Alpharetta, Georgia, United States of America; Michigan State University, United States of America

## Abstract

Maternal nutrition exclusively during the periconceptional period can induce remarkable effects on both oocyte maturation and early embryo development, which in turn can have lifelong consequences. The objective of this study was to evaluate the effect of maternal methionine supplementation on the transcriptome of bovine preimplantation embryos. Holstein cows were randomly assigned to one of two treatments differing in level of dietary methionine (1.89 Met vs. 2.43 Met % of metabolizable protein) from calving until embryo flushing. High quality preimplantation embryos from individual cows were pooled and then analyzed by RNA sequencing. Remarkably, a subtle difference in methionine supplementation in maternal diet was sufficient to cause significant changes in the transcriptome of the embryos. A total of 276 genes out of 10,662 showed differential expression between treatments (FDR <0.10). Interestingly, several of the most significant genes are related to embryonic development (e.g., *VIM*, *IFI6*, *BCL2A1*, and *TBX15*) and immune response (e.g., *NKG7*, *TYROBP*, *SLAMF7*, *LCP1*, and *BLA-DQB*). Likewise, gene set enrichment analysis revealed that several Gene Ontology terms, InterPro entries, and KEGG pathways were enriched (FDR <0.05) with differentially expressed genes involved in embryo development and immune system. The expression of most genes was decreased by maternal methionine supplementation, consistent with reduced transcription of genes with increased methylation of specific genes by increased methionine. Overall, our findings provide evidence that supplementing methionine to dams prior to conception and during the preimplantation period can modulate gene expression in bovine blastocysts. The ramifications of the observed gene expression changes for subsequent development of the pregnancy and physiology of the offspring warrant further investigation in future studies.

## Introduction

Epidemiological studies in humans and experimental studies using animal models have shown that maternal nutrition during different stages of pregnancy can induce permanent changes in the structure, physiology, and metabolism of the offspring [1], [2]. This phenomenon has been termed fetal or developmental programming and could have important implications after birth. Namely, alterations in fetal nutritional status may result in developmental changes, which in turn may predispose the individual to metabolic, endocrine, and cardiovascular diseases in postnatal life [3].

There is increasing evidence that oocyte maturation, fertilization, and preimplantation embryonic development are particularly sensitive periods to changes in maternal nutrition [4]. Indeed, studies in animal models have shown that nutritional changes limited to the period around conception can have both short and long term consequences [5]. For instance, maternal diabetes during oogenesis, fertilization, and the one-cell zygote stage can induce congenital malformations and growth retardation in midgestation mouse embryos [6]. A maternal low protein diet fed during the preimplantation period of rat development caused blastocyst abnormalities and altered birth weight, postnatal growth rate, and adult hypertension [7], [8]. Similarly, a low protein diet administered exclusively during the preimplantation period in mice resulted in increased weight after birth, cardiovascular pathologies, perturbations to renin-angiotensin homeostasis, and abnormal anxiety-related behavior in the offspring [9], [10]. Furthermore, a maternal high fat diet prior to conception is associated with mouse oocytes and zygotes that have altered mitochondrial function including increased membrane potential and biogenesis and increased reactive oxygen species [11]. Overall, these studies are consistent with the idea that maternal nutritional status before and during the periconceptional period can induce remarkable effects on both oocyte maturation and early embryo development, which, in turn, can have lifelong consequences.

Maternal nutrition induces epigenetic alterations in the fetal genome such as DNA methylation, which in turn leads to permanent changes in the phenotype of the offspring [12]. DNA methylation depends on the availability of methyl donors supplied by different amino acids including methionine, and key compounds of the one-carbon metabolism pathways such as choline, betaine, vitamin B12, and folic acid [13]. Some of the most remarkable examples of changing epigenetically sensitive genes via maternal diet are shown with metastable epialleles in mice [14]. Metastable epialleles, such as *agouti viable yellow* and *axin fused*, are alleles that are variably expressed in genetically identical individuals due to epigenetic modifications. In particular, methyl supplements in the diet of pregnant mice have been reported to increase the methylation level of the *agouti* and *axin fused* genes, which consequently led to changes in the coat color (from yellow to brown) and reduction in tail kinking of the offspring, respectively [15], [16]. Studies in ruminants have also shown that changes in methyl donors in the maternal diet during pregnancy can produce epigenetic and physiological changes in the offspring. For example, a recent study in sheep reported that feeding maternal diets rich in methyl group donors increased methylation and altered expression of the imprinted genes *IGF2R* and *H19* in fetal tissues compared to low-methyl group diets [17]. Overall, these studies have clearly established that maternal diet has a transgenerational effect on the offspring through epigenetic modifications.

The decline in reproductive performance of dairy cattle is of major concern to farmers and the dairy industry worldwide. In this sense, methionine supplementation before and around conception seems to be a promising tool for improving reproductive performance in lactating dairy cattle [18]. Previous studies in ruminants have indicated that a methionine-deficient diet provided to the dam prior to and for the first six days after conception can produce substantial changes in the offspring such as elevated blood pressure, insulin resistance, and altered immune response [19]. Since embryos were transferred from the methionine-deficient dams to untreated recipient ewes at 6 days after conception, the effects of the treatment on the future health of the offspring in this experiment occurred prior to Day 6 of pregnancy [19]. Based on these previous results, we hypothesized that a subtle change in methionine status could produce epigenetic changes in the early embryo leading to changes in gene expression by Day 7 in the embryo. Determination of the changes in the transcriptome of the early embryo could foreshadow potential effects of methionine on the future development of the embryo and offspring. As such, the specific objective of this study was to evaluate the effect of methionine supplementation before and until Day 7 after conception on the transcriptome of preimplantation embryos from lactating dairy cows.

## Materials and Methods

### Ethics Statement

This study was approved by the Animal Care and Use Protocol committee of the Research Animal Resources Center at the University of Wisconsin-Madison.

### Treatments and Collection of Embryos

A total of 72 Holstein cows were randomly assigned to one of two treatments differing in level of dietary methionine from calving until embryo flushing (around 70 d postpartum). The treatments were: (1) methionine-rich; diet formulated to deliver 2,875 g metabolizable protein (MP) with 6.8 lysine %MP and 2.4 Met %MP; (2) control; same basal diet but formulated to contain only 1.9 Met %MP. Both diets were formulated to contain 16.5% crude protein (DM basis) and similar concentrations of energy, fiber, starch, fat, macro- and micro-minerals, and vitamins [20]. The methionine-rich diet was supplemented with rumen-protected methionine (Smartamine® M, Adisseo, Atlanta, GA) while the control ration was not. Target levels of MP and methionine were according to NRC [20]. Cows were superovulated with a modified Double Ovsynch protocol [21]. Cows were presynchronized using Ovsynch [22] (GnRH –7 d – PGF2a –3d – GnRH) with a intravaginal progesterone insert (CIDR®, Zoetis Animal Health, NJ, USA) present between the first GnRH and PGF_2α_ of Ovsynch. Seven days after the second GnRH of Ovsynch, cows had all dominant follicles greater than 5 mm aspirated with an ultrasound-guided transvaginal approach (Aloka SSD-900V; 7.5-MHz convex array transducer; Aloka Co., Wallingford, CT, USA) using a 17-gauge X 55 cm aspiration needle. A CIDR device was inserted in all cows after follicular aspiration and superovulation began 35 to 40 h later using FSH treatment equivalent to 400 mg of NIH-FSH-P1 (Folltropin-V) in 8 decreasing doses (3, 3, 2, 2, 1.5, 1.5, 1, 1 ml) administered i.m. at 12 h intervals over a 4 day period. During the superovulatory period, all cows received two PGF_2α_ injections at 48 and 72 hours after CIDR insertion (concomitant with the 5^th^ and 7^th^ FSH injections), and CIDR was withdrawn at 84 hours after insertion (concomitant with the last FSH injection). Twenty-four hours after CIDR withdrawal, ovulation was induced with 3300 IU of human Chorionic Gonadotropin (hCG; Chorulon; Intervet, Millsboro, NJ, USA). Cows were artificially inseminated at 12 and 24 h after hCG using 1 of 2 high-fertility sires. Embryos were flushed 6 days after synchronized ovulations using a transcervical non-surgical technique. A total of 8 cows, 4 per treatment, were then selected, based on the number and quality of the embryos obtained and evenly balanced by sire that was used during AI.

### RNA Extraction and Amplification

Preimplantation embryos recovered from each cow were pooled in order to generate two replicates per cow assayed. Each pool consisted of 1–4 expanded blastocysts with excellent or good quality according to International Embryo Transfer Society standards. Table S1 shows the number of blastocysts used in each sequence sample. A total of 16 embryo pools underwent RNA extraction and amplification using RNaqueous Micro (Life Technologies, Grand Island, NY) and subsequently amplified using the MessageAmp II aRNA amplification kit (Life Technologies). Quantification of amplified RNA was done using the Qubit® 2.0 Fluorometer (Life Technologies) and quality control was done using the *Fragment Analyzer™* Automated CE System (Ames, IA, USA). The concentration and the quality of the amplified RNA are provided in Table S1. Three rounds of linear amplification provided a proper concentration of RNA for subsequent RNA-Seq analysis.

### Library Generation and RNA Sequencing

Libraries of amplified RNA from each pool were prepared following Illumina’s mRNA-Seq protocol. Sequencing libraries were prepared from 50 ng RNA samples and sequenced with Illumina’s HiSeq 2000 at the University of Wisconsin-Madison Biotechnology Center. The 16 libraries were barcoded, multiplexed, and sequenced in two HiSeq 2000 lanes (8 libraries per line). A read was defined as a 100 bp cDNA fragment sequenced from a single end. Approximately 20 million reads were sequenced from each library.

### Mapping Reads to the Reference Genome

Raw sequence reads were mapped directly to the reference genome (bosTau7) using the software package Tophat (v2.0.4) [23]. To maximize sensitivity to splice junction discovery, the first alignment was performed in each of the samples independently. Then, novel splice junctions and known splice junctions from the National Center for Biotechnology Information (NCBI) annotation were combined and supplied to Tophat for a second alignment. This strategy allows a full utilization of the novel junctions identified in the samples. A maximum of 2 mismatches were allowed and reads that mapped equally well to more than 40 genomic locations were discarded.

### Assembly of Transcripts and Estimation of Abundance

The resulting alignments were used to reconstruct transcript models using Cufflinks (v2.0.2) [24]. This program uses graph theory to find a parsimonious set of transcript models that satisfy with the alignments. In addition, the tool *cuffmerge* was used for merging together each of the assemblies with the reference bovine annotation file in order to combine novel isoforms with known isoforms. This procedure maximizes the overall quality of the final assembly [24]. Finally, for each sample, the number of reads that mapped to each gene was obtained with the script *htseq-count* (using mode intersection-nonempty) developed by Simon Anders at EMBL (Genome Biology Unit, Heidelberg, Germany).

### Identification of Differentially Expressed Genes

Genes with total counts per million below 15 were not included in further analysis. Differentially expressed genes were detected using the *edgeR* package (v.3.0.8) [25]. This package uses an overdispersed Poisson model to account for both biological and technical variability. Briefly, *edgeR* combines (1) the application of the trimmed mean of M-values (TMM) as normalization method, (2) an empirical Bayes procedure for estimating the genewise negative binomial dispersions, and (3) an exact test for assessing differential gene expression. Here, all the analyses were performed using the default settings for all parameters. Finally, to account for multiple hypothesis testing and control the false discovery rate, *P*-values reported by the exact test were corrected using the Benjamini-Hochberg procedure [26].

### Validation of Differentially Expressed Genes

Four differentially expressed genes were chosen for validation of RNA-Seq results: vimentin (*VIM*), interferon, alpha-inducible protein 6 (*IFI6*), cytochrome P450, subfamily I (aromatic compound-inducible), polypeptide 1 (*CYP1A1*), and PTC7 protein phosphatase homolog (S. cerevisiae) (*PPTC7*). The RNA samples used for RNA-Seq were used here for validation of differential expression. The cDNA was prepared from each sample using the iScript cDNA synthesis kit (Bio-Rad Laboratories, CA). Individual cDNA samples were diluted 1∶5 and then pooled creating one pool per treatment (i.e., control and methionine-rich treatment). For expression analysis, the reverse-transcriptase PCR (RT-PCR) was used to test the presence or absence of gene expression, whereas quantitative real-time PCR (qRT-PCR) was used to estimate the fold change expression between treatments. The glyceraldehyde-3-phosphate dehydrogenase (*GAPDH*) gene was chosen as internal control because of its stable expression across all RNA-Seq samples. The two cDNA pools were combined with iQSYBR Green Supermix (Bio-Rad Laboratories, CA) and were run in triplicates in the ECO real-time PCR system (Illumina, San Diego, CA). All primers were designed to span exon-exon junctions to minimize the potential of amplifying genomic DNA and are shown in Table 1. The relative gene expression values were calculated using the 2^−ΔΔCt^ method [27].

**Table 1:** Primers used for the validation of gene expression.

Gene	Primer sequence 5′→3′	Amplicon size (bp)
*CYP1A1*	F: ATCCCTGTCCTCCGTTACCT	129
	R: CCGGATGTGACCCTTCTCAAA	
*GAPDH*	F: TGCCCAGAATATCATCCC	134
	R: AGGTCAGATCCACAACAG	
*IFI6*	F: CTCCTCCAAGATACGGTGACAA	120
	R: TTTCGTCTTCCTCCTCGCAG	
*PTC7*	F: CATGAGCTGGCCTATGACCC	88
	R: CTGGCTTTCCACCTCTCACA	
*VIM*	F: GGAAGAGATGGCTCGTCACC	118
	R: AGAAATCCTGCTCTCCTCTCCT	

### Gene Set Enrichment Analysis

The possible enrichment of Gene Ontology (GO) terms, KEGG pathways, and InterPro entries with genes differently expressed between the two treatments was analyzed using a test of proportions based on the cumulative hypergeometric distribution [28]. To avoid testing overly narrow categories, we tested only functional categories with more than 20 genes. Furthermore, the procedure proposed by Benjamini and Hochberg [26] was applied to account for multiple testing. Functional categories with FDR ≤0.05 were considered significant. These analyses were performed using the procedure FatiGO [29], implemented on the platform Babelomics [30].

## Results

### Sequencing of the Transcriptome of Bovine Preimplantation Embryos

To evaluate the effect of maternal methionine supplementation on the transcriptome of bovine preimplantation embryos, 16 embryo pools, 8 pools per treatment, were analyzed using RNA-Seq. Table 2 displays the overall results of sequencing read alignments to the bovine reference genome. Approximately, 20 million reads were sequenced for each sample. Sequencing reads were aligned against the recent bovine reference genome (bosTau7) using the software package Tophat. On average, 75% of the total reads were successfully mapped by allowing no more than two mismatches and restricting the alignments to, at most, 40 genomic locations (Table 2). Importantly, among the aligned reads, over 90% were mapped to unique genomic regions (Table 2). Finally, prior to the statistical analysis, we assessed the number of uniquely mapped reads that were aligned to annotated exons (Table 2). For sample C_3.2_, only 12% of the uniquely mapped reads mapped to annotated exons, i.e. an unexpectedly high number of reads mapped to introns and intergenic regions. Possibly, this was due to an error during the library preparation. Therefore, this sample was not further analyzed. Sequencing data can be accessed by GEO with the accession number GSE48147.

**Table 2:** Summary of sequencing read alignments to the reference genome.

Sample	TotalReads	Total Mapped Reads	Percent Mapped	Uniquely mapped Reads	Percent uniquely mapped	Reads mapped to annotated exons
C_1.1_	18,683,178	14,865,139	80	13,804,648	93	7,579,921
C_1.2_	24,617,256	16,065,170	65	14,644,960	91	6,668,507
C_2.1_	16,999,297	12,754,794	75	11,670,891	92	5,624,126
C_2.2_	19,352,730	14,232,476	74	13,064,631	92	6,461,949
C_3.1_	18,158,624	13,692,675	75	12,524,307	91	7,423,716
C_3.2_	20,488,552	15,917,040	78	14,282,447	90	1,745,048
C_4.1_	17,751,765	14,181,546	80	12,937,347	91	6,066,196
C_4.2_	19,319,918	15,736,767	81	14,532,068	92	7,778,414
M_5.1_	21,048,132	16,307,207	77	14,993,073	92	7,257,007
M_5.2_	18,785,042	14,273,157	76	13,063,772	92	7,578,668
M_6.1_	21,231,578	16,546,777	78	15,056,124	91	7,339,356
M_6.2_	15,886,593	12,745,983	80	11,738,823	92	5,941,973
M_7.1_	19,294,383	14,642,903	76	13,280,140	91	7,826,542
M_7.2_	15,078,732	11,628,448	77	10,663,643	92	6,582,004
M_8.1_	18,057,137	14,080,144	78	12,768,027	91	7,184,256
M_8.2_	19,600,350	14,327,542	73	12,981,732	91	7,405,798

Samples belong to control (C) and methionine-rich (M) treatments, respectively. A total of 8 cows, 4 per treatment, were used. Embryos recovered from each cow were pooled in order to generate two replicates per cow assayed.

### Exploration, Evaluation, and Validation of Overall Gene Expression

**Figure 1:**
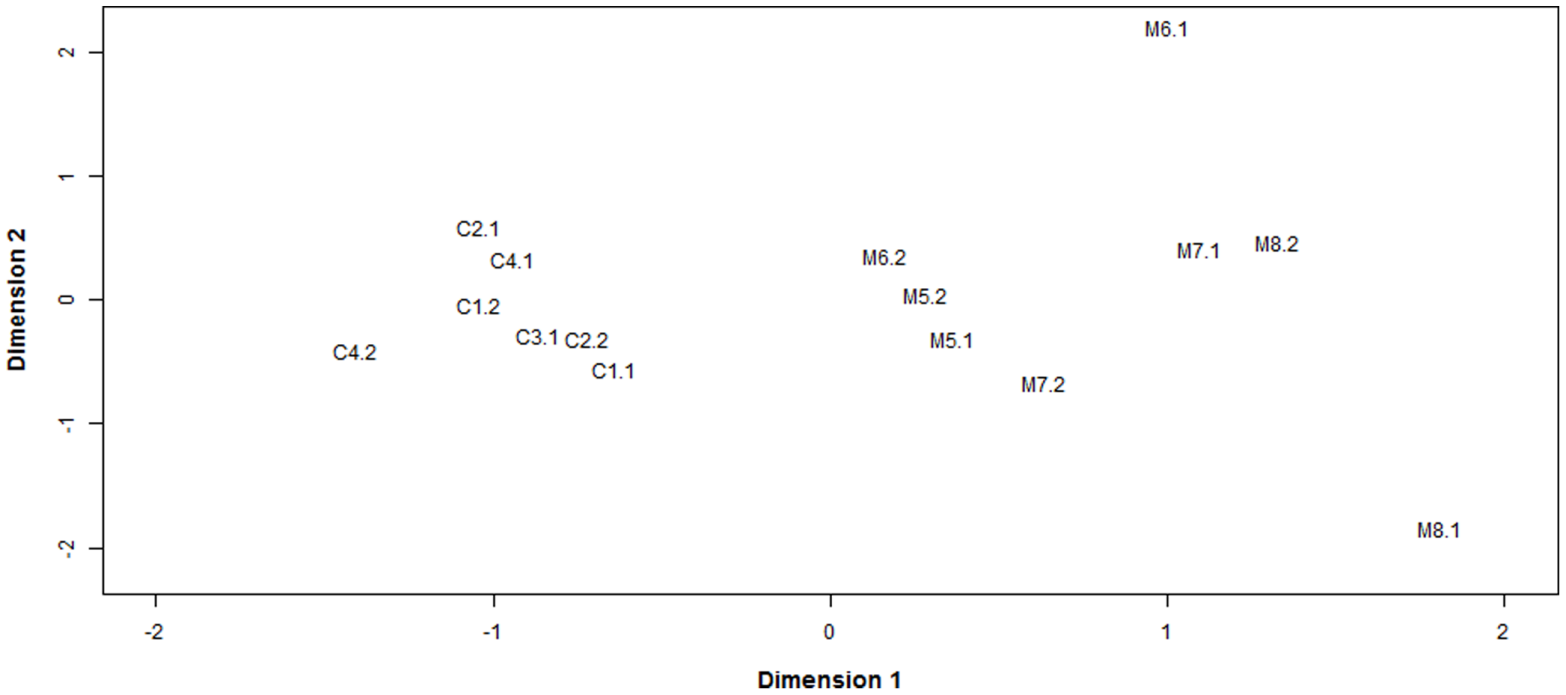
Multidimensional scaling (MDS) plot showing the relative similarities of the samples under study. Distance between each pair of samples is the square root of the common dispersion based on the top 1,000 genes that best distinguished that pair of samples.

To visualize the overall relationship between the samples, a multidimensional scaling (MDS) analysis was performed (Figure 1). Basically, an MDS plot shows the relative similarities of the samples under study. Here, the distance between each pair of samples is the square root of the common dispersion based on the 1,000 genes with most heterogeneous expression, i.e. top 1,000 genes that best distinguished each pair of samples. Interestingly, the MDS plot showed that dimension 1 clearly separated the control from the methionine samples (Figure 1). This implies that the biological replicates are consistent and that multiple differentially-expressed genes between treatments can be detected.

**Figure 2:**
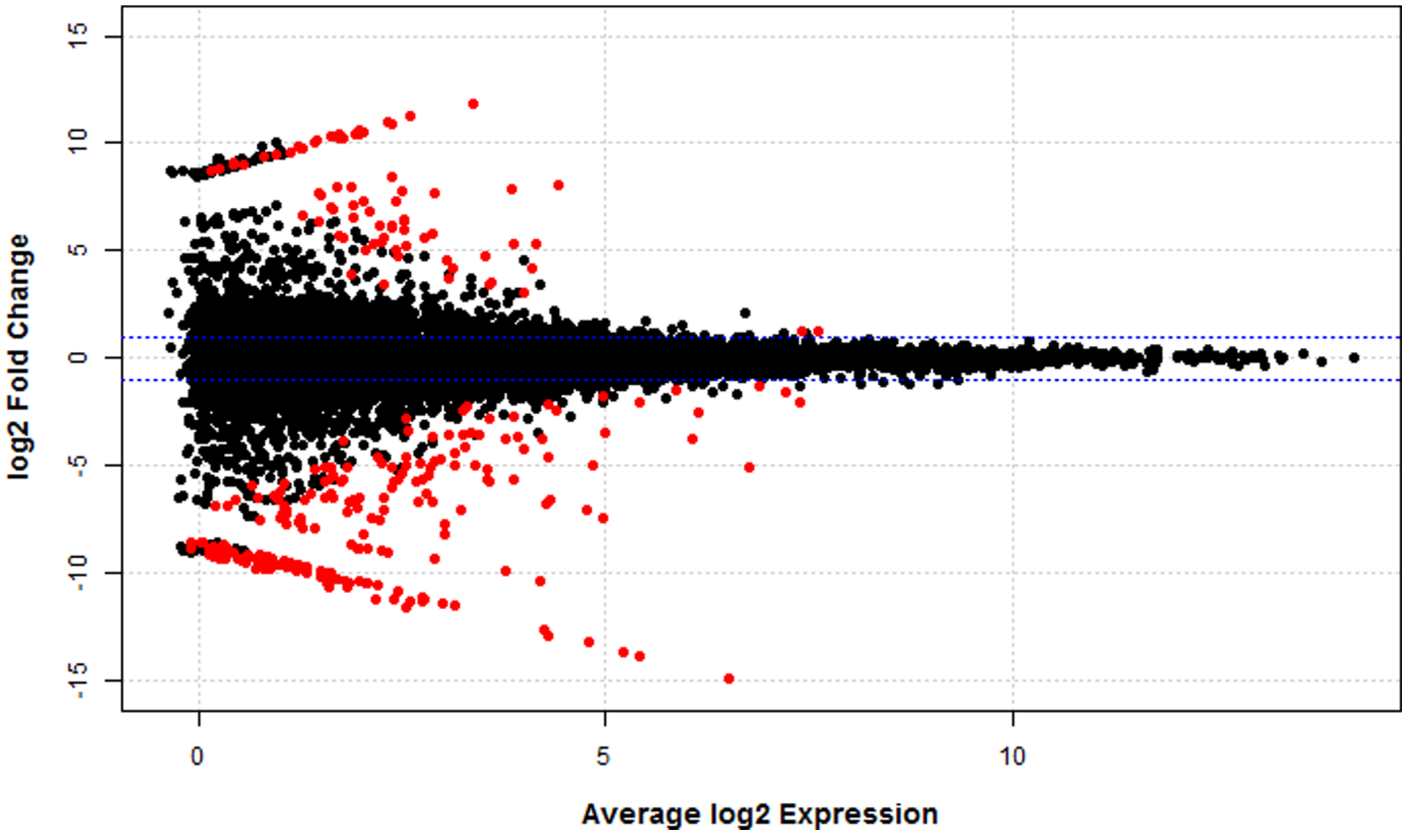
Plot of the log2 fold change against average log2 expression. Gene expression was expressed as counts per million. Horizontal blue lines indicate 2-fold change. Differential expressed genes (FDR <0.10) are highlighted in red.

A total of 10,662 genes were tested for differential expression. Controlling false discovery rate (FDR) at 0.10, a total of 276 genes showed at least a 2-fold expression difference between treatments. In particular, 200 genes showed higher expression in the control treatment while 76 genes showed higher expression in the methionine-rich treatment. Figure 2 displays the log2 fold change (i.e., the log2 of the ratio of expression levels for each gene between the two experimental groups) against the average log2 expression (i.e., the overall average expression level for each gene across the two experimental groups). As expected, most of the genes showed a fold difference very close to 0. Additionally, differentially expressed genes seem to be evenly distributed in the range between low and moderate abundance levels (Figure 2).

Table 3 shows the top 30 most statistically significant genes (FDR <0.0035). Twenty-eight of these 30 genes showed greater expression in control samples compared with samples from the methionine-rich treatment. Notably, many of these genes are directly involved in embryo development, such as vimentin (*VIM*), interferon, alpha-inducible protein 6 (*IFI6*), BCL2-related protein A1 (*BCL2A1*), and T-box 15 (*TBX15*). In addition, several of these 30 genes are related to immune response. For instance, natural killer cell group 7 sequence (*NKG7*), TYRO protein tyrosine kinase binding protein (*TYROBP*), signaling lymphocyte-activating molecule family 7 family member 7 (*SLAMF7*), lymphocyte cytosolic protein 1 (*LCP1*), BoLa class II histocompatibility antigen (*BLA-DQB*), and SHC (Src homology 2 domain containing) transforming protein 2 (*SHC2*), are genes closely related to innate and adaptive immune responses. Moreover, genes associated with insulin receptor signaling pathway, e.g. insulin receptor (*INSR*) and eukaryotic translation initiation factor 4E family member 1B (*EIF4E1B*), showed differential expression between treatments. Finally, some uncharacterized genes and also novel transcript units were among the most significant genes. Both novel transcript units CUFF.2147.1 (chr29∶45,539,922-45,543,303) and CUFF.606.1 (chr13∶42,645,356-42,673,392) were supported by known cattle ESTs from the UCSC genome browser [http://genome.ucsc.edu/]. This is further evidence that not only are there uncharacterized regions of the bovine genome, but also that these regions may serve important biological roles.

**Table 3:** Top 30 most significant genes that showed differential expression between control and methionine-rich treatment.

Gene	Name	log2 FC	FDR
LAPTM5	Lysosomal protein transmembrane 5	−14.9	4.7×10^−9^
NKG7	Natural killer cell group 7 sequence	−13.6	4.4×10^−8^
VIM	Vimentin	−13.8	1.8×10^−7^
TYROBP	TYRO protein tyrosine kinase binding protein	−13.2	3.2×10^−6^
IFI6	Interferon, alpha-inducible protein 6	−12.6	1.5×10^−5^
CUFF.2147.1	*Novel transcript unit*	−8.2	1.5×10^−5^
LOC505451	Olfactory receptor, family 1, subfamily J, member 2-like	−13.0	1.5×10^−5^
SLAMF7	Signaling lymphocyte-activating molecule family 7 family member 7	−10.4	3.5×10^−5^
LOC788199	Olfactory receptor 6C74-like	−10.4	7.6×10^−5^
LCP1	Lymphocyte cytosolic protein 1 (L-plastin)	−9.9	1.1×10^−4^
LOC100849660	*Uncharacterized*	11.9	2.2×10^−4^
BLA-DQB	MHC class II antigen	−11.1	2.2×10^−4^
SHC2	SHC (Src homology 2 domain containing) transforming protein 2	−11.5	3.4×10^−4^
NT5C3	5′-nucleotidase, cytosolic III	−11.5	3.5×10^−4^
LOC510193	Apolipoprotein L, 3-like	7.8	4.3×10^−4^
LOC100848815	SLA class II histocompatibility antigen, DQ haplotype D alpha chain-like	−11.4	4.3×10^−4^
CUFF.606.1	*Novel transcript unit*	−5.6	4.3×10^−4^
LOC100850656	*Uncharacterized*	−11.2	4.8×10^−4^
SLC11A1	Solute carrier family 11 (proton-coupled divalent metal ion transporters), member 1	−10.7	6.9×10^−4^
LOC100852347	Beta-defensin 10-like	−11.2	7.3×10^−4^
LOC100297676	C-type lectin domain family 2 member G-like	−6.8	9.2×10^−4^
BCL2A1	BCL2-related protein A1	−7.1	1.2×10^−3^
INSR	Insulin receptor	−5.1	1.3×10^−3^
NOVA1	Neuro-oncological ventral antigen 1	−10.6	1.5×10^−3^
TBX15	T-box 15	−11.2	2.2×10^−3^
TMEM200C	Transmembrane protein 200C	−6.6	2.2×10^−3^
GPNMB	Glycoprotein (transmembrane) nmb	−7.5	2.3×10^−3^
ARHGAP9	Rho GTPase activating protein 9	−5.7	2.7×10^−3^
EIF4E1B	Eukaryotic translation initiation factor 4E family member 1B	−11.3	3.1×10^−3^
LOC100295170	Protein BEX2-like	−9.3	3.5×10^−3^

A negative log2 Fold Change (FC) value means that the gene showed higher expression in control treatment while a positive value means that the gene showed higher expression in methionine-rich treatment.

**Figure 3:**
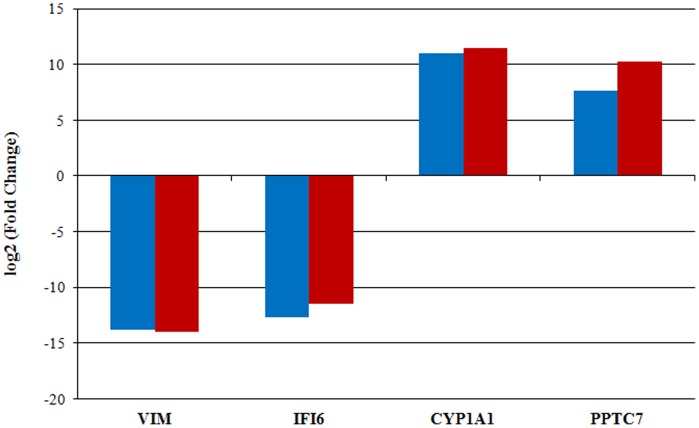
Fold changes of four differentially expressed genes measured by RNA-Seq (blue) versus qRT-PCR (red). Bars above the X-axis denote genes with higher expression in the methionine-rich treatment while bars below the X-axis denote genes with higher expression in the in control treatment. Importantly, IFI6 was not expressed in methionine-rich pool and hence a fold change was assigned based on the detection threshold of the equipment.

To validate genes found to be significant in the RNA-Seq analysis, 4 differentially expressed genes (*VIM*, *IFI6*, *CYP1A1*, and *PPTC7*) were selected and their expression was assessed using PCR-based methods. RNA-Seq analysis revealed that *VIM* and *IFI6* were exclusively expressed in control embryos, while *CYP1A1* and *PPTC7* were highly expressed in methionine-rich embryos. The absence of *IFI6* expression in methionine-rich embryos was validated using RT-PCR. The remaining three genes were validated using qRT-PCR. Figure 3 displays the fold differences in gene expression measured by RNA-Seq and the PCR-based methods. Overall, these 4 genes showed similar patterns of mRNA abundance with both methods (Figure 3).

### Gene Set Enrichment Analysis: Evaluation of Different Gene Ontology Terms, KEGG Pathways, and InterPro Motifs

To gain insight into the processes that could be regulated differentially between control and methionine-supplemented embryos, we performed a gene set enrichment analysis. Genes that showed a FDR <0.20 and had ENSEMBL annotations (n = 335) were tested against the background set of all genes with ENSEMBL annotations (n = 8,848). We found 33 GO terms significantly enriched (FDR <0.05) with differentially expressed genes (Table 4 and Table S2). Noticeably, many of these functional categories are directly associated with embryo development, e.g. *Tube development* (GO:0035295), *Branching morphogenesis of a tube* (GO:0048754), and *Morphogenesis of a branching structure* (GO:0001763). Additionally, several GO terms are closely related to immune response. For instance, *Defense response* (GO: 0006952), *Immune response* (GO:0006955), *Response to wounding* (GO:0009611), *Cytokine binding* (GO:0019955), and *Cytokine receptor binding* (GO:0005126) were significantly enriched with genes differentially expressed between treatments. Many GO terms related to transmembrane receptor/transporter activity were also significant. Furthermore, 12 InterPro entries showed significant overrepresentation (FDR <0.05) of differentially expressed genes (Table S3). Interestingly, several of these terms are associated with immunoglobulin and cytochrome P450 motifs. Finally, one KEGG pathway, *cytokine-cytokine receptor interaction* (bta04060), was also significant (FDR = 0.018).

**Table 4:** Gene Ontology (GO) biological process terms significantly enriched with differentially expressed genes.

GO ID	Term	FDR (q-value)
0001763	Morphogenesis of a branching structure	0.038
0006952	Defense response	0.004
0006955	Immune response	0.038
0007166	Cell surface receptor linked signal transduction	0.023
0007186	G-protein coupled receptor protein signaling pathway	0.010
0009611	Response to wounding	0.025
0035295	Tube development	0.038
0048754	Branching morphogenesis of a tube	0.037

To gain more details on the possible effect of maternal methionine supplementation on the transcriptome of the embryos, we further characterized the most relevant pathways in order to assess the general pattern of gene expression within these terms. In the case of GO term *Tube development*, 8 of the 11 significant genes were decreased in the methionine-rich treatment. Moreover, 15 genes of the GO term *Immune response* showed differential expression between treatments. Interestingly, 14 of these genes were decreased in the embryos from the methionine-rich treatment. Additionally, 10 out of 13 differentially-expressed genes for the InterPro motif *Immunoglobulin-like* (IPR007110), and 8 out of 10 differentially-expressed genes for KEGG pathway *Cytokine-cytokine receptor interaction* (bta04060), were decreased in methionine-supplemented embryos compared to control embryos. Table S4 provides a list of the significant genes (using Ensembl gene ID) in each of these functional categories.

## Discussion

It is well established that nutritional perturbations exclusively during the periconceptional period can modify development throughout gestation and even affect the physiological and metabolic health of adult offspring [4], [5]. Of particular interest to this research, a previous study that restricted methyl donors by restricting methionine, vitamin B12, and folate before and for the first 6 days after conception, demonstrated that normally-appearing embryos on Day 6 after conception can produce offspring with substantial differences in subsequent physiology [19]. The present study was specifically designed to determine whether methionine supplementation during follicular development and early embryo development, until Day 7, modifies gene expression of the early embryo. Remarkably, a subtle increase of methionine in maternal diet caused notable changes in the transcriptome of preimplantation embryos. Our findings support the idea that oocyte maturation, fertilization, and preimplantation embryonic development are particularly sensitive periods to changes in maternal nutrition.

Although the embryos in both treatments possessed very similar morphological appearance and received the same rating for developmental stage and embryo quality (expanded blastocyst with excellent quality), they showed significant transcriptomic differences. In fact, normally appearing early embryos have been found to have substantial differences in gene expression profiles [31] and produce offspring with substantial differences in physiology [19]. In our study, 276 of the 10,662 genes analyzed showed significant differences between treatments (fold change >2.0 and FDR <0.10). Interestingly, expression of 72% of the significant genes was decreased in the embryos derived from dams fed a methionine-rich diet. This result is consistent with the expected effect on the fetal genome of an increase in methionine in the maternal diet. Increased levels of methyl donors, such as methionine, in the one-carbon pathway would increase DNA methylation levels of many fetal genes, which in turn could suppress gene expression. Methylation of DNA generally is thought to produce transcriptional repression, particularly when methylation occurs at CpG islands associated with the promoter of a particular gene [32]. However, methylation of CpG islands in the 3′ region of certain genes has been recently linked with increased transcription of those genes [32]. Important insights into epigenetic regulation are provided by the studies of metastable epialleles in mice [33]. Metastable epialleles, such as *agouti viable yellow* (*A^vy^*) and *axin fused*, are very susceptible to environmental effects. For example, in the *A^vy^* mice, dams fed the control NIH-31 diet described as “methyl-sufficient” during pregnancy, produced offspring with insufficient DNA methylation of the *A^vy^* allele during pregnancy, leading to improper overexpression of this allele in ectopic tissues, and eventually causing the yellow obese mouse syndrome [15]. Supplementation of the dams with mid-range or high-methyl supplementation, including supplemental methionine, increased methylation of the *A^vy^* allele, reducing abnormal, ectopic *A^vy^* expression, and reduced expression of the yellow obese mouse phenotype [15]. Although DNA methylation was not directly evaluated in our experiment, it seems likely that methionine supplementation increased methylation of key regions of DNA and this change probably underlies the methionine-associated changes in the transcriptome of the Day 7 bovine embryo. This link between maternal diet and subsequent modification of gene expression in the embryonic genome is one of the important molecular mechanisms proposed to explain the phenomenon of fetal programming [1], [3], [12].

The precise effects of methionine supplementation on reproductive efficiency of dairy cattle and on physiology of the offspring have not yet been adequately evaluated. This is in spite of the wide-spread use of methionine supplementation in the world-wide dairy industry. Of special interest, we found that many of the most significantly altered genes are associated with early embryo development. For instance, *VIM* encodes a member of the intermediate filaments and its expression is essential for normal development of bovine embryos [34]. *IFI6*, one of the genes induced by interferon with a critical role in the regulation of apoptosis, is expressed differentially as part of the early endometrial response after conception and is considered a marker of a viable preimplantation embryo in cattle [35]. *BCL2A1* encodes a member of the BCL2 protein family which plays a critical role in the regulation of oocyte and early embryo survival [36]. *TBX15* belongs to the T-box family of genes, a family of transcription factors that are involved in early embryo development, regulation of the development of extraembryonic structures, and many aspects of organogenesis [37]. However, it is intriguing to investigate whether the substantial decrease in expression of these genes found in embryos from methionine-supplemented cows is likely to be positive or negative for subsequent embryo survival and embryo development. For example, *VIM* is known to be expressed during bovine gastrulation (Day 14) and development of the primitive streak (Day 21) but was not observed by immunocytochemistry in the bovine embryo before hatching and elongation [34]. Similarly, *VIM* expression in the porcine embryo, as detected by immunohistochemistry, is only observed after hatching and early elongation of the porcine embryo [38]. Thus, methionine-induced suppression of *VIM* in the Day 7 embryo could be viewed in a similar light as the methyl-induced repression of the *A^vy^* gene in agouti mice, as suppressing inappropriate gene expression and allowing subsequent appropriate expression in a cell-specific manner. Thus, our experimental design allowed us to distinguish key changes in the transcriptome of the early embryo but these changes should be interpreted with caution since the proper timing and cellular localization of mRNA expression is critical for optimal embryonic development and physiology, as elegantly demonstrated in previous studies of the *agouti* gene in mice.

One specific example of the difficulty in interpreting gene expression results is the marked down-regulation of genes associated with the GO term *Tube development* in embryos from dams supplemented with methionine. Neural tube development occurs shortly after implantation and hence a maternal diet rich in methionine may affect this essential process in cattle. However, suboptimal circulating folate and methionine concentrations, two critical components of the methyl-donor pathway, are associated with neural tube defects in many mammals including humans. In fact, epidemiological and experimental studies have indicated that periconceptional supplementation of components of the methyl donor pathway, including folate and methionine, can prevent ∼70% of neural tube defects [39]–[41]. Thus, suppression of gene expression in the *Tube development* pathway, as found with methionine supplementation in our study, could be positive for subsequent embryonic and fetal gene expression based on observations that supplementation of methyl donors in diets during early pregnancy improves neural tube development in most animal and human studies.

Of particular interest, maternal methionine supplementation had significant effects on the expression of genes and pathways that are important for the innate and adaptive immune responses in adults. For instance, the *NKG7* gene encodes a cell surface protein expressed in several cell types, including natural killer cells and active T cells [42]. *TYROBP*, a transmembrane protein initially characterized in natural killer cells, is involved in a broad array of biological functions, including the innate response against viruses, and diverse inflammatory reactions mediated by neutrophils, monocytes and macrophages [43]. *SLAMF7* is involved in the activation of natural killer cells and also in the proliferation of B lymphocytes during innate and adaptive immune responses [44]. *LCP1*, a leukocyte-specific F-actin bundling protein, is implicated in T lymphocyte polarity and migration in response to chemokines [45]. *BLA-DQB* is one of the genes of the major histocompatibility complex in cattle, a family of highly polymorphic genes that play a central role antigen presentation during adaptive immune responses [46]. Importantly, all these genes were decreased in the embryos from methionine-supplemented dams. Furthermore, several significant pathways related to immune and defense response, such as GO terms *Immune response*, *Defense response*, and *Cytokine binding*, KEGG pathway *Cytokine-cytokine receptor interaction*, and several *Immunoglobulin like* InterPro motifs were significantly enriched with differentially expressed genes. Notably, all these pathways were down-regulated in embryos from methionine-supplemented dams. Again, it is not clear whether decreased expression of these genes in the early embryo will be associated with improvements or decreases in specific aspects of immune function in the future offspring. A previous study in sheep, showed that methionine changes in the diet of dams during the periconceptional period, led to male lambs that had a reduced serum haptoglobulin response to ovalbumin challenge [19]. In mice, Hollingsworth et al. [47] reported that a maternal diet rich in methyl donor groups affect T lymphocyte maturation and development in mice. In particular, fetal exposure to methyl donor groups modifies T lymphocyte cytokine production favoring lymphocyte maturation into a Th2 phenotype [47]. In addition, the authors found that key regulatory genes that control the development of an adaptive immune response were differentially methylated, and interestingly, these methylation changes were associated with decreased transcriptional activity [47]. Overall, our results provide further evidence that a maternal diet rich in methyl donor groups may alter the expression of genes associated with immune response. Obviously, Day 7 embryos do not yet have an immune system, and therefore early embryonic expression of these genes could be viewed as aberrant or as an important preparatory step for subsequent embryonic differentiation. Importantly, these alterations could have long-term implications on the survival and health of the offspring, which warrants further research.

In summary, our study has characterized the effect of supplemental methionine during the periconceptional period on the transcriptome of the bovine preimplantation embryo. Expressions of many genes that are critical for subsequent embryonic and adult function are decreased by methionine supplementation, probably due to increased DNA methylation in CpG islands in the promoters of these genes. Determination of the effect of methionine supplementation on specific DNA methylation patterns in these genes is needed in future studies. In addition, the expression of a few transcripts was increased in the Day 7 embryo by methionine supplementation of the dam, an intriguing observation that should be investigated in the future. Of particular importance, the functional ramifications of the observed changes in gene expression, in terms of immune response, pregnancy progression, and physiology of the offspring, warrant substantial future investigation.

## Supporting Information

Table S1 **Number of blastocysts pooled in each sample, and concentration and quality of the amplified RNA used for subsequent sequencing.**
(DOC)

Table S2 **Gene Ontology (GO) cellular component and molecular function terms significantly enriched with differentially expressed genes.**
(DOC)

Table S3 **InterPro motifs significantly enriched with differentially expressed genes.**
(DOC)

Table S4 **List of significant genes in the most remarkable functional categories.**
(DOC)
