# Supplementary material for: Effect of Maternal Methionine Supplementation on the Transcriptome of Bovine Preimplantation Embryos
Source: PLoS One. 2013 Aug 21;8(8):e72302. doi: 10.1371/journal.pone.0072302 (PMC3749122; doi:10.1371/journal.pone.0072302)
Supplement: Table S1 — Number of blastocysts pooled in each sample, and concentration and quality of the amplified RNA used for subsequent sequencing. (DOC) [file pone.0072302.s001.doc]

**Table S1. Number of blastocysts pooled in each sample, and concentration and quality of the amplified RNA used for subsequent sequencing**

| **Sample** | **Number of blastocysts** | **Total Concentration (ng/μL)** | **RNA Quality Number** |
| --- | --- | --- | --- |
| C_1.1_ | 2 | 88.5 | 8.6 |
| C_1.2_ | 2 | 70.9 | 8.9 |
| C_2.1_ | 2 | 120.9 | 9.0 |
| C_2.2_ | 2 | 480.8 | NA |
| C_3.1_ | 2 | 132.3 | 8.9 |
| C_3.2_ | 2 | 16.5 | 10.0 |
| C_4.1_ | 1 | 52.0 | 8.6 |
| C_4.2_ | 1 | 200.0 | NA |
| M_5.1_ | 3 | 261.7 | 7.2 |
| M_5.2_ | 3 | 256.0 | 6.9 |
| M_6.1_ | 3 | 29.1 | 8.0 |
| M_6.2_ | 4 | 26.6 | 7.9 |
| M_7.1_ | 2 | 48.3 | 6.6 |
| M_7.2_ | 3 | 65.3 | 8.9 |
| M_8.1_ | 2 | 14.3 | 8.9 |
| M_8.2_ | 1 | 8.0 | 10.0 |

Samples belong to control (C) and methionine-rich (M) treatments. A total of 8 cows, 4 per treatment, were used. Embryos recovered from each cow were pooled in order to generate two replicates per cow assayed.
