# Supplementary material for: Effect of Maternal Methionine Supplementation on the Transcriptome of Bovine Preimplantation Embryos
Source: PLoS One. 2013 Aug 21;8(8):e72302. doi: 10.1371/journal.pone.0072302 (PMC3749122; doi:10.1371/journal.pone.0072302)
Supplement: Table S2 — Gene Ontology (GO) cellular component and molecular function terms significantly enriched with differentially expressed genes. (DOC) [file pone.0072302.s002.doc]

**Table S2. Gene Ontology (GO) cellular component and molecular function terms significantly enriched with differentially expressed genes**

| **GO ID** | **Term** | **FDR (q-value)** |
| --- | --- | --- |
| **Cellular Component** | | |
| 0005615 | Extracellular space | 0.041 |
| 0005886 | Plasma membrane | 0.041 |
| 0016021 | Integral to membrane | 0.017 |
| 0044459 | Plasma membrane part | 0.033 |
| **Molecular Function** | | |
| 0003700 | Transcription factor activity | 0.040 |
| 0004197 | Cysteine-type endopeptidase activity | 0.026 |
| 0004497 | Monooxygenase activity | 0.024 |
| 0004871 | Signal transducer activity | 0.001 |
| 0004872 | Receptor activity | < 0.001 |
| 0004888 | Transmembrane receptor activity | 0.040 |
| 0004930 | G-protein coupled receptor activity | < 0.001 |
| 0005102 | Receptor binding | 0.002 |
| 0005126 | Cytokine receptor binding | 0.008 |
| 0005216 | Ion channel activity | 0.048 |
| 0005509 | Calcium ion binding | 0.013 |
| 0008234 | Cation transmembrane transporter activity | 0.036 |
| 0008528 | Peptide receptor activity, G-protein coupled | 0.006 |
| 0015075 | Ion transmembrane transporter activity | 0.040 |
| 0015267 | Channel activity | 0.049 |
| 0019955 | Cytokine binding | 0.006 |
| 0022857 | Transmembrane transporter activity | 0.040 |
| 0022891 | Substrate-specific transmembrane transporter activity | 0.036 |
| 0030246 | Carbohydrate binding | 0.007 |
| 0030247 | Polysaccharide binding | 0.012 |
| 0042277 | Peptide binding | < 0.001 |
