# Supplementary material for: Effect of Maternal Methionine Supplementation on the Transcriptome of Bovine Preimplantation Embryos
Source: PLoS One. 2013 Aug 21;8(8):e72302. doi: 10.1371/journal.pone.0072302 (PMC3749122; doi:10.1371/journal.pone.0072302)
Supplement: Table S3 — InterPro motifs significantly enriched with differentially expressed genes. (DOC) [file pone.0072302.s003.doc]

**Table S3. InterPro motifs significantly enriched with differentially expressed genes**

| **InterPro** | **Term** | **FDR (q-value)** |
| --- | --- | --- |
| 001671 | Melanocortin / ACTH receptor | 0.043 |
| 002286 | P2 purinoreceptor | 0.043 |
| 002397 | Cytochrome P450, B-class | 0.041 |
| 002401 | Cytochrome P450, E-class, group I | 0.043 |
| 002403 | Cytochrome P450, E-class, group IV | 0.043 |
| 003596 | Immunoglobulin V-set, subgroup | 0.043 |
| 003598 | Immunoglobulin subtype 2 | 0.043 |
| 003599 | Immunoglobulin subtype | 0.043 |
| 006052 | Tumor Necrosis Factor | 0.043 |
| 007110 | Immunoglobulin-like | 0.035 |
| 008967 | p53-like transcription factor, DNA-binding | 0.035 |
| 013106 | Immunoglobulin V-set | 0.035 |
