# Supplementary material for: Effect of Maternal Methionine Supplementation on the Transcriptome of Bovine Preimplantation Embryos
Source: PLoS One. 2013 Aug 21;8(8):e72302. doi: 10.1371/journal.pone.0072302 (PMC3749122; doi:10.1371/journal.pone.0072302)
Supplement: Table S4 — List of significant genes in the most remarkable functional categories. (DOC) [file pone.0072302.s004.doc]

**Table S4. List of significant genes in the most remarkable functional categories:** GO categories *Tube development* (GO:0035295) and *Immune response* (GO:0006955), InterPro term Immunoglobulin-like (IPR007110), and KEGG pathway Cytokine-cytokine receptor interaction (bta04060).

**GO:0035295**

ENSBTAG00000019302, ENSBTAG00000015718, ENSBTAG00000011578, ENSBTAG00000001060, ENSBTAG00000005691, ENSBTAG00000016915, ENSBTAG00000012465, ENSBTAG00000003636, ENSBTAG00000025029, ENSBTAG00000017541, ENSBTAG00000015460

**GO:0006955**

ENSBTAG00000019302, ENSBTAG00000019588, ENSBTAG00000000977, ENSBTAG00000015718, ENSBTAG00000007191, ENSBTAG00000021466, ENSBTAG00000027513, ENSBTAG00000003895, ENSBTAG00000037605, ENSBTAG00000016683, ENSBTAG00000025471, ENSBTAG00000012223, ENSBTAG00000025782

**IPR007110**

ENSBTAG00000019588, ENSBTAG00000000977, ENSBTAG00000001235, ENSBTAG00000031430, ENSBTAG00000020046, ENSBTAG00000033225, ENSBTAG00000015457, ENSBTAG00000016915, ENSBTAG00000003176, ENSBTAG00000037605, ENSBTAG00000004355, ENSBTAG00000001197, ENSBTAG00000010458

**bta04060**

ENSBTAG00000007191, ENSBTAG00000027513, ENSBTAG00000001060, ENSBTAG00000038045, ENSBTAG00000005910, ENSBTAG00000025471, ENSBTAG00000012223, ENSBTAG00000025782
